# ALL IN THE FAMILY: DIFFERENTIAL IMPACT OF COVID-19 BY TYPE OF CAREGIVER FOR PERSONS LIVING WITH DEMENTIA

**DOI:** 10.1093/geroni/igad104.0348

**Published:** 2023-12-21

**Authors:** Amanda Leggett, Sophia Tsuker

**Affiliations:** Wayne State University, Detroit, Michigan, United States; Wayne State University, Detroit, Michigan, United States

## Abstract

Existing research points toward higher burden for spousal, and particularly wife, caregivers for persons living with dementia (PLwD). Yet how relationship roles and stressors shifted during the COVID-19 pandemic, where some care dyads were isolated together, or physically distancing, is unknown. The current study explores how the variability in reports of COVID-specific care-related stress (e.g., worry about the PLwD getting COVID-19) and general strain (e.g., caregiver burden) differed among husband, wife, adult child, and other family and non-family, unpaid caregivers. Participants include 100 primary family caregivers for PLwDs (mean age=63.3 years, 75% female) from the COVID STYLE study of which 22 were husbands, 39 were wives, 24 were adult children, and 15 were another relation (e.g., daughter-in-law, friend). ANOVAs and linear regressions (adjusting for cohabitation with the PLwD, dementia severity, and race) were run with each relation as a predictor of COVID-19-specific stress (e.g., sleep quality, isolation) and non-COVID-19-specific well-being (e.g., depressive symptoms, positive affect). The “other” caregiver group showed significantly greater COVID-related financial difficulty of care, worse sleep quality, and isolation, whereas adult children were most worried about the PLwD getting COVID-19 and had greater difficulty in their ability to provide care. Associations largely held in adjusted regressions showing particular contrast between other caregivers and husbands, who fared best. On the other hand, relationships groups did not differ on non-COVID-specific measures of well-being. Non-spousal caregivers reported greater pandemic stress related to caregiving, suggesting an overlooked group who may have provided significant COVID-19 care despite a more distant relationship.

